# How Much of Language Acquisition Does Operant Conditioning Explain?

**DOI:** 10.3389/fpsyg.2017.01918

**Published:** 2017-10-31

**Authors:** Christopher B. Sturdy, Elena Nicoladis

**Affiliations:** ^1^Neuroscience and Mental Health Institute, Department of Psychology, University of Alberta, Edmonton, AB, Canada; ^2^Department of Psychology, University of Alberta, Edmonton, AB, Canada

**Keywords:** operant conditioning, language learning, imitation, ontogenetic ritualization, language socialization

## Abstract

Since the 1950s, when Chomsky argued that Skinner’s arguments could not explain syntactic acquisition, psychologists have generally avoided explicitly invoking operant or instrumental conditioning as a learning mechanism for language among human children. In this article, we argue that this is a mistake. We focus on research that has been done on language learning in human infants and toddlers in order to illustrate our points. Researchers have ended up inventing learning mechanisms that, in actual practice, not only resemble but also in fact are examples of operant conditioning (OC) by any other name they select. We argue that language acquisition researchers should proceed by first ruling out OC before invoking alternative learning mechanisms. While it is possible that OC cannot explain all of the language acquisition, simple learning mechanisms that work across species may have some explanatory power in children’s language learning.

## Chomsky vs. Skinner

Skinner (1957) argued that language acquisition could be explained by mechanisms of operant conditioning (OC). OC is a technique that can be used to target and increase a behavior by pairing performance of the target behavior with a positive or rewarding outcome (Domjan, 2010). Chomsky (1959) rebutted Skinner’s argument on several grounds, including that it was unlikely that parents were doing the slow and careful shaping of children’s vocalizations and that there are grammatical regularities that cannot be discerned from the surface features of language alone (e.g., the connection between statements and questions that involve different word order). Textbooks on language development often present the Skinner–Chomsky debate as if Chomsky won. Consider this example as evidence: “a child could not possibly learn through imitating all the sentences she or he has the potential of producing later. Nor could a child experience all possible sentences in order to become aware of successive word associations, as Skinner suggested” (Owens, 2008, p. 33). Another line of reasoning sometimes used to dismiss OC as a learning mechanism for language is that parents do not consistently provide feedback to children about the grammaticality of their language use and, even if they do, children often ignore the feedback (Owens, 2008). Skinner did, admittedly, overstate his case. For example, Skinner (1957) expounded on a verbal behavioral explanation of a Shakespearean sonnet (pp. 350–351), without having articulated all the logical steps between parents reinforcing infants’ vocalizations and sonnets.

The argument of this paper is that developmentalists have been too hasty in dismissing Skinner’s approach to language acquisition. By denying themselves access to basic principles of OC, they sometimes construct language learning mechanisms *de novo*. These learning mechanisms are basically OC but researchers have called them by other names, such as language socialization and ontogenetic ritualization. In doing so, researchers remove the connection to a long and rich history of research on learning in both humans and non-human animals. We conclude that language acquisition researchers should first attempt to use these well-understood learning mechanisms. It is possible that OC will not explain all aspects of language acquisition yet we argue that only when OC has been shown to be an insufficient explanation should we devise new learning mechanisms. To illustrate our points, we focus on language learning in infancy and toddlerhood.

## Studies Framed in a Learning Perspective

Before showing how researchers have only superficially dismissed OC as a learning mechanism, it is important to acknowledge that some research is framed in a learning perspective (e.g., Goldstein et al., 2003, 2009). In an attempt to demonstrate similarities between songbird vocal learning and human speech learning, Goldstein et al. (2003) designed an experiment testing the impact of social reinforcement on vocal production of human infants. This experiment was inspired by earlier work with songbirds and was an attempt to create an analogous situation for infants.

Songbirds are a non-human model of choice for experimental work aimed at understanding the mechanisms of vocal learning. This is in large part because songbirds learn their songs during development in a manner analogous to human speech acquisition and because vocal learning, production, and perception is subserved by an increasingly well-defined set of brain areas, also analogous to human speech learning, production, and perception.

In many species, song acquisition is based on vocal imitation such that young males imitate the songs of nearby tutors (for an overview, see Catchpole and Slater, 2008). In some species, such as brown-headed cowbirds (*Molothrus ater*), song learning is impacted by other factors beyond imitation of a tutor song. For instance, work by West and King (1988) showed that female cowbirds produced a “wing stroke,” a movement of their wing, following the production of preferred songs. In this way, females of this songbird species were signaling or reinforcing males’ production of particular songs. Later, when females were tested with wingstroke and non-preferred songs, wingstroke songs led to more precopulatory displays, thus linking their reinforcement of the wingstroke songs to their preferences. Based on what was known about songbird vocal learning, Goldstein et al. (2003) designed a human analog of vocal learning through reinforcement.

The design was elegant in its simplicity. Mothers and 8-month-old infants were tested in one of the two conditions. Both conditions had a similar procedure: 30-min familiarization phase, 10-min baseline monitoring, 10-min intervention phase, and 10-min extinction phase. What differed between the two conditions was the details of the intervention phase. Both groups were told to respond socially, but not verbally, by smiling and moving toward the infants. In the contingent condition, mothers made these responses contingent with infants’ vocalizations, while the yoked group made these responses in a non-contingent manner. Thus, mothers from both groups behaved similarly with only one group doing so contingent on vocal output by their infant (i.e., reinforcing). The result was that contingent-condition mothers socially reinforced vocal output by their infants, whereas the yoked mothers did not. Vocal output was quantified and compared between the groups in a number of ways that assessed the amount and quality of vocalizations. The results of intervention were significant and striking with the infants in the contingent condition producing more and higher quality vocalizations than the infants in the yoked condition. Thus, this work provides a clear example of contingent social feedback acting as a reinforcer, leading to an increase in vocal output and quality of vocalizations produced. Essentially, the interpretation is that infants in the contingent condition increased their vocalizations because their vocalizations were followed by a reinforcing event, the contingent social feedback.

## Current Proposed Language Learning Mechanisms

In this section, we discuss three language learning mechanisms researchers have proposed for children’s early language acquisition: language socialization, ontogenetic ritualization, and imitation. We show that these are compatible with OC.

### Language Socialization

To address Chomsky’s (1965) claim that language was unlearnable from messy input, some researchers pointed out that parents can simplify the language presented to children, using a register sometimes called baby talk or caregiverese (Gleitman et al., 1984; Fernald, 1985). If this register was universal, then young children might initially learn only a simple subset of their input language(s). Once that subset was well learned, children could progress to more complex language. Research from anthropologists brought into question the universality of this register: Schieffelin and Ochs (1986) showed that parents in Western Samoa did not simplify the language presented to young children. In other cultures, parents do not address their children directly until the children start to speak (Schieffelin and Ochs, 1986). Nevertheless, all typically developing children end up speaking.

In order to account for the cross-cultural variability in how parents address their children, Schieffelin and Ochs (1986; Ochs, 1993) argued that children learn language by being socialized into the culture-specific ways of behaving, including language use. The attributes of language socialization are as follows: bidirectional socialization, the use of a special register to address young children, asymmetrical social relations, cross-cultural differences in how parents talk with children, parental treatment of children as cultural members even before the knowledge of how to act appropriately as a cultural member is the children’s own, and finally that children understand their own and others’ identity through language socialization (Schieffelin and Ochs, 1986). All attributes but the last (about identity) of language socialization are perfectly compatible with OC.

Within learning theory, any behavior that results in a reinforcer, whether primary like food, drugs, or sex or secondary such as feedback from a parent teaching a child language, results in the targeted response being strengthened and more likely to occur in the future (e.g., Domjan, 2010). This phenomenon was first demonstrated by Thorndike (1898) who timed cats’ escape latencies from a puzzle box that was reinforced with access to fish. What he showed was that latencies decreased over trials. While Thorndike (1898) focused on the cats’ behavior, it is possible to conceptualize this learning as bidirectional (i.e., the cats have also taught the experimenters to give them fish). Nonetheless, the usual conceptualization of learning studies is that there is a tutor and a tutee, in other words, that there are asymmetrical relations. As for the parents treating children as cultural members, this may be another way of saying that children respond to social reinforcers. For social animals, like humans and birds, it is unsurprising that social interactions reinforce children’s behavior (Kang et al., 2013).

There is nothing within a learning framework that would necessarily involve a special language register used to address children or that would predict cross-cultural differences in how parents interact with children. There is, however, nothing within a learning perspective that would be incompatible with these characteristics. In fact, the observation that babies pay particular attention to caregiverese (Singh et al., 2002) could be taken as evidence that babies condition their parents to speak to them in that register.

The fact that social identity is not compatible with a learning perspective is not sufficient to dismiss our interpretation of language socialization as fundamentally OC for two reasons. First, social identity is thought to emerge developmentally from how children act and are treated (Ochs, 1993). In other words, social identity may not be a definitional part of language socialization, but rather a common outcome of its implementation. Second, language socialization researchers studying infants and toddlers have generally not included identity as a component under study. Including identity as a variable in connection with language socialization is more typically done in studies with adolescents (e.g., Caldas and Caron-Caldas, 2002).

If researchers deliberately took a learning perspective in studying the effect of interlocutors on language acquisition, some current results that are incompatible across studies might be clarified. Consider the following example: bilingual children do not necessarily use the language the parents do or code-switch to the same extent parents do (e.g., Pan, 1995). In one language socialization study, Lanza (1992) argued that bilingual children might code-switch more with parents who respond more like bilinguals (e.g., indicate their comprehension of the other language) than with parents who respond more like monolinguals (e.g., act mystified when the child uses the “wrong” language). Lanza (1992) showed that this explanation worked within a Norwegian-English bilingual family. Other studies with other bilingual children have found similar results (Genesee et al., 1996; Lanza, 1998; Juan-Garau and Perez-Vidal, 2001). Indeed, advice to parents of bilingual children often includes how they should respond to the undesired language choice (Kohnert et al., 2005). However, one study showed that Lanza’s (1992) explanation could not be extended to French-English bilingual children in Montreal (Nicoladis and Genesee, 1998). It is possible that longitudinal studies of the children’s learning histories could help explain why different bilingual children respond differently to the very same kind of parental language use (see, for example, Mishina-Mori, 2011).

By deliberately adopting a learning perspective, researchers can articulate other testable hypotheses based on well-documented characteristics about learning. For example, as noted earlier, Thorndike (1898) showed that latencies in response time decreased over the course of learning. If this finding extends to humans, one prediction that follows is that children’s latency to respond to similar social situations should decrease as they get older. Future research can systematically test that possibility in the context of language learning.

### Ontogenetic Ritualization

Another learning mechanism that has been invoked to explain some of infants’ early communicative symbols is ontogenetic ritualization (Clark, 1978; Lock, 1978; Tomasello, 2006). Ontogenetic ritualization refers to a gradual process of learning, with the learner initially a relatively passive participant in an action with another human being (usually a parent). Children and parents gradually learn to anticipate each other’s actions and produce their part of the action even before the cue. When they can produce their part before the cue, children can understand that that action has communicative value in and of itself and can therefore use it symbolically (e.g., as a request or demand). Ontogenetic ritualization has been proposed as the developmental origin of the “give” gesture and the “pick-me-up” gesture (Lock, 1978; Tomasello, 2006). In the case of the former, infants would initially participate in the action of reaching; in the latter, the action of being picked up. Some researchers claim that ontogenetic ritualization is also the origin of gestures in non-human primates (Call and Tomasello, 2007; cf. Genty et al., 2009).

Researchers have implied that ontogenetic ritualization differs from basic learning principles because each participant has a specified role within a dyad and the actions originate in goal-directed activity and show a large amount of individual variation (Tomasello et al., 1997). However, these characteristics are all compatible with OC. As noted earlier, according to OC, the tutor and the tutee have well-defined roles: the tutor provides feedback in the form of reinforcement in a manner contingent with the tutee’s behavior. There is nothing about OC that would preclude goal-directed activity and individual variation is commonly reported among studies using OC (e.g., Guillette et al., 2011).

Furthermore, empirical studies of human infants have shown that basic learning mechanisms are sufficient to explain children’s learning of some communicative gestures. Marentette and Nicoladis (2012) examined the interactions of parents and infants between 6 and 12 months to test for ontogenetic ritualization as the developmental origin of “give” and “pick-me-up” gestures. They showed that parents gave positive social reinforcement for these gestures, such as smiling, giving the child something or picking up the child. While some infants were already producing instances of these gestures by the age of 6 months, there is evidence for earlier learned actions as the basis of these gestures. Reddy et al. (2013) showed that even younger infants learn through basic learning mechanisms to shift their bodies in anticipation of being picked up.

In addition, researchers originally introduced the construct of ontogenetic ritualization to explain early symbolic acquisition in primates. However, there is little reason to think that ontogenetic ritualization differs definitionally from OC. More importantly, the empirical evidence to date has generally shown that OC is sufficient to explain the acquisition of early symbols.

### Imitation

Language acquisition researchers generally agree that imitation of ritualized actions is one important mechanism for acquiring language and other cultural norms (Tomasello, 2009; Rossano, 2012). Children actively participate in ritualized actions starting around 9 to 12 months of age (Tomasello, 2009) and usually produce their first words while doing ritualized actions (Bloom, 2000). Imitation has been unambiguously included as part of basic learning mechanisms for several decades (Bandura, 1965) although it has not always been clear how imitation might be reinforced in human interactions. Recently, Ray and Heyes (2011) suggested one possibility: they argued that imitation could be learned by infants being imitated themselves (in other words, through social reinforcement).

Two aspects of human imitation may initially seem outside of the explanatory reach of OC. First, language acquisition researchers have shown that children can produce novel linguistic constructions from fairly early in development (Tomasello, 1992). However, these novel constructions are often small variations from constructions they have heard before (Tomasello, 1992, 2000; Lieven et al., 2003). While OC has been caricaturized as restricting children to produce the exact sentences they have heard before (see opening paragraph), there is extensive evidence that even infants can show generalization in their associative learning, if only through forgetting the original stimuli (Vlach, 2014).

A second aspect of human imitation that may seem outside of OC is selective social learning. Children do not randomly imitate: they imitate some people more than others (Poulin-Dubois and Brosseau-Liard, 2016). Poulin-Dubois and Brosseau-Liard (2016) argue that children selectively imitate knowledgeable and trustworthy models. An interesting direction of future research is whether children use their learning histories with particular people to decide who is knowledgeable and trustworthy. For example, it may turn out that children are more likely to imitate people who give consistent social feedback.

## Conclusion and Future Directions

We have argued here that despite deliberate efforts, developmentalists have not, in practice, rid themselves of basic learning mechanisms like OC in explaining language acquisition. Instead, they have often invoked learning mechanisms that restate the basic conditions for reinforcement learning. As noted earlier, Goldstein et al. (2003) showed how only 10 min of social reinforcement can have dramatic effects on vocal output in infants. One can only imagine the role that such patterns of reinforcement play during the entire developmental period of a childhood.

We would like to be clear about we are not claiming *a priori* that basic learning mechanisms can explain all aspects of language acquisition (or even Shakespearean sonnets). There may be human-specific learning mechanisms required to explain some aspects of language acquisition. For example, maybe such learning mechanisms are necessary to explain how English-speaking children learn to form questions (see the discussion in Chomsky, 1965). Furthermore, it is entirely possible that multiple learning mechanisms interact with each other so that children learn language.

Our point is, instead, a procedural one. We are suggesting that we should see how far OC gets us before seeking new human-specific learning mechanisms. For example, it is possible that learning to form questions from statements is explainable within an OC framework. As noted earlier, there is increasing evidence that children’s earliest signs of productive syntax are in the form of small changes from previous constructions. Children’s gradual emergence of productive syntax could be due to social reinforcement from parents. In any case, it is important to empirically rule out OC as a learning mechanism for productive syntax. By embracing this procedure, developmentalists can benefit from the long history of research on learning to make predictions. To do this research seriously, observing language acquisition *in situ*, over the course of a child’s development, is one important approach. This approach is rapidly becoming technologically feasible (Roy and Pentland, 2002). Another important approach involves experimental intervention, including training and extinction schedules, following well-studied learning procedures (e.g., Goldstein et al., 2003).

In conclusion, let us not throw out the baby with the bathwater: simple learning mechanisms that work across species may have some explanatory power in children’s language learning.

## Author Contributions

CS and EN jointly contributed to the conceptualization and writing of this article.

## Conflict of Interest Statement

The authors declare that the research was conducted in the absence of any commercial or financial relationships that could be construed as a potential conflict of interest.
